# The effect of high polycyclic aromatic hydrocarbon exposure on biological aging indicators

**DOI:** 10.1186/s12940-023-00975-y

**Published:** 2023-03-17

**Authors:** Manuela Campisi, Giuseppe Mastrangelo, Danuta Mielżyńska-Švach, Mirjam Hoxha, Valentina Bollati, Andrea A. Baccarelli, Angela Carta, Stefano Porru, Sofia Pavanello

**Affiliations:** 1 Occupational Medicine, Department of Cardio-Thoraco-Vascular Sciences and Public Health, University of Padua, Padua, Italy; 2 Faculty of Medical Sciences Prof. Zbigniew Religa, Silesian Academy, Zabrze, Polska; 3 Epidemiology, Epigenetics and Toxicology Lab, Dipartimento Di Scienze Cliniche E Di Comunità, Università Degli Studi Di Milano, Milan, Italia; 4 UO Epidemiologia, Fondazione IRCCS Ca’ Granda Ospedale Maggiore Policlinico, Milan, Italia; 5 Department of Environmental Health Sciences, Mailman School of Public Health, Columbia University, New York, NY USA; 6 Department of Diagnostics and Public Health, University of Verona and Clinical Unit of Occupational Medicine, University Hospital of Verona, 37134 Verona, Italy; 7 Padua Hospital, Occupational Medicine Unit, Padua, Italy; 8 University Center for Space Studies and Activities “Giuseppe Colombo” - CISAS. University of Padua, Padua, Italy

**Keywords:** Biological aging, DNA alterations, DNA methylation age, Mitochondrial DNA copy number, Occupational exposure, Polycyclic aromatic hydrocarbons, Structural equation modelling, Telomere length

## Abstract

**Background:**

Aging represents a serious health and socioeconomic concern for our society. However, not all people age in the same way and air pollution has been shown to largely impact this process. We explored whether polycyclic aromatic hydrocarbons (PAHs), excellent fossil and wood burning tracers, accelerate biological aging detected by lymphocytes DNA methylation age (DNAmAge) and telomere length (TL), early nuclear DNA (nDNA) hallmarks of non-mitotic and mitotic cellular aging, and mitochondrial DNA copy number (mtDNAcn).

**Methods:**

The study population consisted of 49 male noncurrent-smoking coke-oven workers and 44 matched controls. Occupational and environmental sources of PAH exposures were evaluated by structured questionnaire and internal dose (urinary 1-pyrenol). We estimated Occup_PAHs, the product of 1-pyrenol and years of employment as coke-oven workers, and Environ_PAHs, from multiple items (diet, indoor and outdoor). Biological aging was determined by DNAmAge, via pyrosequencing, and by TL and mtDNAcn, via quantitative polymerase chain reaction. Genomic instability markers in lymphocytes as target dose [anti-benzo[a]pyrene diolepoxide (anti-BPDE)–DNA adduct], genetic instability (micronuclei), gene-specific (p53, IL6 and HIC1) and global (Alu and LINE-1 repeats) DNA methylation, and genetic polymorphisms (GSTM1) were also evaluated in the latent variable nDNA_changes. Structural equation modelling (SEM) analysis evaluated these multifaceted relationships.

**Results:**

In univariate analysis, biological aging was higher in coke-oven workers than controls as detected by higher percentage of subjects with biological age older than chronological age (AgeAcc ≥ 0, *p* = 0.007) and TL (*p* = 0.038), mtDNAcn was instead similar. Genomic instability, i.e., genotoxic and epigenetic alterations (LINE-1, p53 and Alu) and latent variable nDNA_changes were higher in workers (*p* < 0.001). In SEM analysis, DNAmAge and TL were positively correlated with Occup_PAHs (*p* < 0.0001). Instead, mtDNAcn is positively correlated with the latent variable nDNA_changes (*p* < 0.0001) which is in turn triggered by Occup_PAHs and Environ_PAHs.

**Conclusions:**

Occupational PAHs exposure influences DNAmAge and TL, suggesting that PAHs target both non-mitotic and mitotic mechanisms and made coke-oven workers biologically older. Also, differences in mtDNAcn, which is modified through nDNA alterations, triggered by environmental and occupational PAH exposure, suggested a nuclear-mitochondrial core-axis of aging. By decreasing this risky gerontogenic exposure, biological aging and the consequent age-related diseases could be prevented.

## Introduction

Aging is associated with higher risk of chronic conditions, such as cardiovascular, cancer and neurodegenerative diseases, and represents a serious health and socioeconomic societal concern [1]. However, not all people become elderly in the same manner [2]. One of the major issues arisen in recent years is that environmental pollution can accelerate aging [3]. Air pollution, jointly with genetic and life-style risk factors, including tobacco smoking, alcohol drinking and unsafe diet, contributes significantly to aging [4, 5]. Air pollution exposure can shorten life expectancy even among people with the best genetic makeup.

Oxidative stress and the consequent chronic inflammation have been identified as pathogenic factors in aging and age-related diseases, partly caused by chemical metabolism, which results in excessive production of oxygen-derived free radicals and low-grade inflammation [6]. The toxicology screening program Tox21, testing biological responses to more than 9,000 chemicals, showed that numerous organic chemicals, including polycyclic aromatic hydrocarbons (PAHs), alter molecular signaling pathways related to inflammation which contribute to the pathogenesis of many age-related diseases [7]. Benzo(a)pyrene (B[a]P) metabolic activation, via aldo–keto reductase and/or manganese superoxide dismutase [8], produces reactive oxygen species (ROS) that can generate high levels of oxidized guanine in nDNA [9]. In our previous work, the exposure to PAHs of coke-oven workers, amongst the highest exposure to these carcinogenic compounds, monitored by internal dose (urinary 1-pyrenol) and by genotoxic measures at target dose [anti-benzo[a]pyrene diolepoxide (anti-BPDE)–DNA adduct]**,** was found to increase a systemic inflammatory response [10] and induced gene-specific (p53, IL6 and HIC1) and global (extrapolated from Alu and LINE-1 repeats) DNA methylation changes [11]. Furthermore, those coke-oven workers with shorter telomere length (TL) [12] showed lower mitochondrial DNA copy number (mtDNAcn), thus linking PAHs exposure and mitochondrial dysfunction with cellular aging [13]. Moreover, an important link between telomeres and mitochondria was made by Sahin et al. [14] with the suggestion of a telomere-mitochondrial core-axis of aging, including tumor suppressor p53 as key regulator.

In the present study, we hypothesized that exposure to higher levels of PAHs in coke-oven workers may be also associated with increased biological aging detected by lymphocyte DNA methylation age (DNAmAge). DNAmAge is an emerging and most robust epigenetic marker of non-mitotic cellular aging [15, 16], assessed through the analysis of methylation at a specific subset of cytosine-guanine dyads (CpG), which showed a strong correlation with the chronological age [17–20]. This biomarker was linked to the “epigenetic clock” theory of aging [21] according to which an increase in DNAmAge is indicative of altered biological functions [16] and an elevated risk for morbidity and mortality [22]. Furthermore, the close nexus we found between DNAmAge of the pulmonary cells and blood lymphocytes, advise that blood lymphocytes could be a surrogate tissue for lung age status [23].

To this purpose, we investigated the effects of high chronic exposure to PAHs on DNAmAge of Polish male, non-current smoking, coke-oven workers and matched controls. We also investigated the relative magnitude of other measures of aging, including TL, which measures mitotic or replicative cellular aging, and mtDNAcn. These multifaceted relationships are evaluated using the structural equation models (SEM) analysis. SEM is a statistical technique linking observed data with qualitative causative assumptions and testing whether variables are interdependent, and if so, the details of their relationships. This methodology is appropriate for the investigation of complex interrelationships, as it tests causative relationships instead of mere correlations [24].

## Materials and methods

### Study design

This is a cross-sectional study comparing a group with high occupational exposure to PAHs and a reference group without such exposure. The study population consisted of *n* = 49 coke-oven workers in 3 Polish cokeries and *n* = 45 controls matched by gender and ethnicity, who were part of a group of 94 study individuals examined in our previous work [12] for whom DNA was still available. All participants were males and noncurrent smokers defined as either never-smokers or former smokers who had quit smoking at least 1 year before sample collection, as confirmed by analysis of nicotine and its metabolites [25]. All coke-oven workers performed tasks (i.e., charging, coking, and pushing operations at the coke-oven battery section) involving exposure to high levels of PAHs. Individuals whose work involved exposure to benzene (i.e., workers in byproduct operations) were excluded. Controls were clerks of the Institute of Occupational Medicine and Environmental Health in Sosnowiec, recruited during their periodic check-ups at the Preventive Health Services of the Institute.

PAH exposure was assessed by measuring 1-pyrenol in a urine sample (50 mL) collected from each of the workers at the end of their work shift (after at least 3 consecutive working days) and in the late afternoon from controls. Simultaneously, blood samples were collected in EDTA (20 mL) and heparin tubes (10 mL) for adduct and micronuclei analyses, as described previously [25], and further genetic [12, 26] and epigenetic analyses. All samples were transported at the Institute of Occupational Medicine and Environmental Health in Sosnowiec where: (i) lymphocytes cultures for micronuclei detection were prepared and micronuclei analyses were conducted; (ii) lymphocytes for adduct analyses were isolated in Ficoll solution (Seromed) within 4 h after blood collection and kept frozen at − 80 °C until shipment to the Occupational Medicine section of the University of Padova, Italy, where DNA was extracted. Structured questionnaires were administered to collect data on other non-occupational PAHs exposure (indoor and outdoor exposure and diet) and daily consumption of fruit or vegetables, as well as information on chronic diseases, as described previously [12, 25].

The Ethics Committee of the Institute of Occupational Medicine and Environmental Health in Sosnowiec reviewed the study. All study participants gave their written informed consent before recruiting.

### Estimation of PAHs exposure from the questionnaire

Structured questionnaires were administered to collect data on non-occupational PAHs exposure, focusing on the following categories.

- Diet. Individuals who declared to consummate PAHs-rich meals (charcoaled meat and pizza) more than once a week were recorded as individuals with high dietary intake of PAHs;
- Indoor. Individuals who declared to use wood or coal heating at home were considered as individuals with indoor exposure to PAHs.
- Outdoor. Individuals who declared to live (home residence) in areas with intense local traffic and/or the presence of industries were considered as individuals with environmental exposure to PAHs.
- Home. Individuals who declared to have the residence in country or town.

### Internal exposure: 1-pyrenol analysis

Exposure to PAHs was determined as previously described [25] by measuring 1-pyrenol in urine samples by high-performance liquid chromatography/fluorescence. The urine sample is pretreated by enzymatic hydrolysis overnight in the dark at 37 °C with 1.25 µl/ml urine of the enzymatic mixture β-glucuronidase (134.8U/ml) and aryl sulfatase (4.0U/ml) (Sigma-Aldrich). 1-pyrenol level in each urine sample was expressed as micromoles per mole of creatinine, determined using a colometric test, based on the Jaffé reaction between creatinine and sodium picrate. 1-pyrenol was multiplied per years of work in the cokery.

### Analysis of nDNA alterations

#### Target dose: anti-BPDE–DNA adduct

*Anti*-BPDE–DNA adduct formation was detected after DNA isolation with a Promega Wizard genomic DNA purification kit (Promega) by high-performance liquid chromatography/fluorescence analysis of BP-tetrol-I-1 (*r*-7,*c*-10,*t*-8,*t*-9-tetrahydroxy-7,8,9,10-tetrahydro-benzo[*a*]pyrene) released after acid hydrolysis of DNA samples, as described previously [25]. The mean coefficient of variation (CV) for analyses of a standard curve repeated five times on five different days was 10%. The highest CV value was 5.70% for those samples (*n* = 8) with more than 200 μg DNA, repeated twice.

#### Genetic instability: micronuclei

Micronuclei analysis was conducted on coded slides scored by light microscopy at × 400 magnification, as described previously [25]. To exclude artifacts, the identification of micronuclei was confirmed at × 1,000 magnification in 10% of samples. The scoring of bi-, tri- and tetra-nucleate cells and micronuclei analysis was done and the cytokinesis block proliferation index was calculated as being equal in both groups (*p* = 0.60).

#### Analyses of DNA methylation states of p53, p16, HIC1 and IL-6 gene-specific promoters and of Alu and LINE-1 repetitive element

DNA methylation status was quantified as previously described [11], using bisulfite-PCR and pyrosequencing. The degree of methylation was expressed as the percentage of methylated cytosines divided by the amount of both methylated and unmethylated cytosines. All samples were analyzed 3 times for each marker to verify the reproducibility of our measurements and their average was used in the statistical analysis.

### Analyses of biological aging indicators

#### mtDNAcn analysis

MtDNAcn was measured in DNA using real-time quantitative PCR (qPCR) as previously described [13, 27]. This assay measures relative mtDNAcn by determining the ratio of mitochondrial (MT) copy number to single copy gene (S) copy number (human β-globin, hbg) in experimental samples relative to the MT/S ratio of a reference pooled sample. The primers for qPCR analysis of mtDNA and hbg were previously described [28]. All qPCRs were performed on 7900HT Fast Real-Time PCR System (Applied Biosystems, Monza, Italy). The average of the three MT measurements was divided by the average of the three S measurements to calculate the MT/S ratio for each sample. The CV for the MT/S ratio in duplicate samples analyzed on two different days was 7.8%.

#### TL analysis

TL was measured in DNA using the qPCR method as described previously [29, 30]. This method appraises the relative telomere length in genomic DNA by establishing the ratio of telomere repeat copy number (T) to single copy gene (S) copy number (T/S ratio) in experimental samples relative to the T/S ratio of a reference pooled sample [29, 30]. The single-copy gene used in this study was human β-globin (hbg). All samples and standards were run in triplicate. The qPCR runs were conducted in triplicate on a 7900HT Fast Real Time PCR System (Applied Biosystems, Monza, Italy) and the average of the 3 T/S ratio measurements was used in the statistical analyses. To test the reproducibility of telomere length measurements, we replicated TL analysis 3 times in 3 different days for 15 samples. The within-sample CV for the average T/S ratio over the 3 consecutive days was 8.7%, which was similar to the CV reported for the original protocol [31].

#### DNAmAge analysis

DNAmAge was assessed by analyzing the methylation levels from specific CpG sites using bisulfite-PCR and Pyrosequencing® methodology as previously reported [32, 33]. This method is based on the determination of the percentage of methylation level measured in five selected markers (ELOVL2, C1orf132, KLF14, TRIM59 and FHL2) in genomic DNA, as described by Zbieć-Piekarska et al. [19] with some modifications based on the fact that the method was almost completely automated using the PyroMark Q48 Autoprep (QIAGEN, Milano, Italy). Briefly, 1 μg DNA was bisulfite treated using Epitect Fast® DNA Bisulfite Kit (QIAGEN, Milano, Italy) following the manufacturer’s instructions. An aliquot of template DNA was used for PCR amplification of five specific markers using PCR primers of the AgePlex Mono kit (Biovectis, Warszawa, Poland). PCR reactions were performed in 25 μL, comprising 0.2 μM of each primer, 20 ng of template DNA, and PyroMark PCR Master Mix holding HotStarTaq DNA Polymerase, 1X PyroMark PCR Buffer and dNTPs. The amplification plan involved a preliminary denaturation step at 95 °C for 10 min, followed by 45 cycles of denaturation (94 °C for 30 s), annealing (56 °C for 60 s) and extension (72 °C for 90 s), with a final extension of 72 °C for 10 min. Every PCR amplification contained negative PCR controls. In total, 10 µL of PCR product was used for each pyrosequencing primer (2 µL) included in the AgePlex Mono kit (Biovectis, Warszawa, Poland) and loaded into a 48 well-plate (Pyromark Q48 Discs, QIAGEN, Milano, Italy). Pyrosequencing was performed on a Pyromark Q48 Autoprep instrument (QIAGEN, Milano, Italy) using Pyromark Q48 Advanced Reagents (QIAGEN, Milano, Italy) according to the manufacturer’s instructions. The resulting Pyrograms® generated by the instrument were automatically analyzed using Pyromark Q48 Autoprep Software (QIAGEN, Milano, Italy). The percentages of marker methylation levels were put in an online calculator system accessible at www.agecalculator.ies.krakow.pl, for estimation of DNAmAge. The equation corresponds to a previously developed age prediction model [19]. All samples were tested 3 times for each marker to confirm the reproducibility of our results, and their averages were used in the statistical testing. All samples were analyzed on two different days, and the CV for replicate pyrosequencing runs was 0.5%.

#### Age acceleration evaluation

Age Acceleration (AgeAcc) was computed for each participant as the difference between the DNAmAge of blood lymphocytes and the chronological age (equation AgeAcc = DNAmAge—Chronological age).

### Statistical analysis

#### Analytic strategy

- Variables

As stated above, chronic exposure to PAHs was twofold, originating from occupation or environmental sources. Occupational exposure to PAHs (Occup_PAHs) was assessed as the product of internal dose (urinary 1-pyrenol) and years of employment as coke workers. This value represents a cumulative occupational exposure that was always zero in control subjects. While occupational PAHs exposure was measured, environmental PAHs exposure was estimated from multiple items (diet and indoor and outdoor living characteristics and activities). The large number of measured covariates called for dimension reduction to avoid over-fitting and collinearity issues in estimating exposure effects [34]. This was obtained by summarizing high-dimensional data in a single summary score, a latent variable named with the descriptive term Environ_PAHs.

There was interest to contrast the effects of environmental and occupational PAH exposure. Therefore, the relative importance of each source was evaluated by forcing both PAH variables into the same model of mediation analysis (see below), where they acted as independent variables.

There were three dependent variables – DNAmAge, TL and mtDNAcn – each being evaluated separately using three models of mediation analysis.

Upon metabolic activation, catalyzed mainly by the cytochrome P450 enzymes, some PAHs become metabolites that react with DNA leading to genotoxic and/or epigenetic alterations. Many biomarkers reflecting the interaction between the external agent and the exposed body are usually included in a broad and heterogeneous category [35]. Therefore, we pooled the available covariates in a second latent variable called "nDNA_changes" (see below). The nature of the latent variable is intrinsically related to the nature of the indicator variables used to define it. The indicator variables were: peripheral blood lymphocytes measures of target dose (anti-BPDE–DNA adducts), genetic instability (micronuclei—MN), DNA methylation (methylation states of p53, p16, HIC1 and IL-6 gene-specific promoters and global methylation estimated in Alu and LINE-1 repeats) and presence of the detoxifying GSTM1. Again, summarizing high-dimensional data in a single summary score allowed for avoiding over-fitting and collinearity issues in regression analyses [34]. That score was particularly useful to act as a mediator variable through which some of the effects of the independent variable pass on to the dependent variable (this is known as the indirect effect).

b) Directed acyclic graph (DAG)

**Fig. 1:**
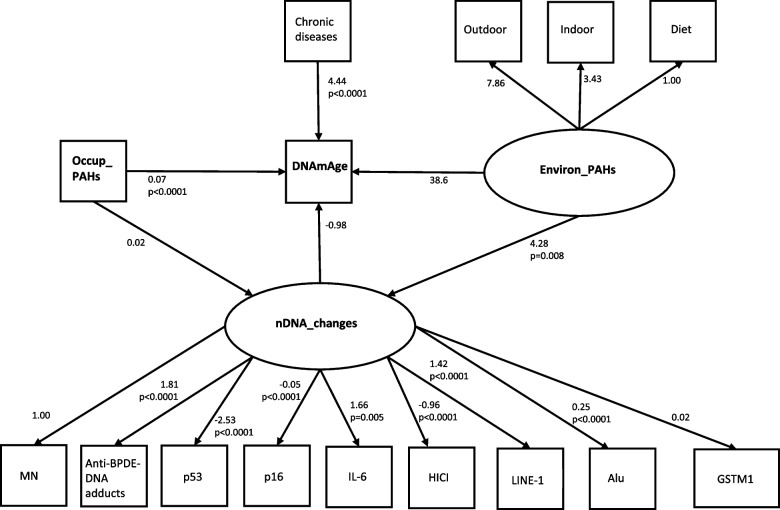
Path diagram of mediation model for DNAmAge. Path diagram of a mediation model showing the dependent variable «DNAmAge», the mediator variable «nDNA_changes» and two independent variables «Occup_PAHs» and «Environ_PAHs». An oval indicates the latent variable, square boxes indicate the observed variables, arrows specify the direction of causal flow, an arrowed route is a path, and the estimated beta coefficients with p-values appeared along the paths. The effect of one variable on another is called direct. Paths from each latent variable (nDNA_changes, Environ_PAHs) to each endogenous variable are also drawn. Abbreviations: DNAmAge = DNA methylation age; nDNA_changes = Latent variable estimated by SEM (overall change of DNA) based on MN, Anti-BPDE-DNA adducts, p53, p16, IL-6, HICI, LINE-1, Alu, GSTM1; Environ_PAHs = Latent variable estimated by SEM (environmental exposure to PAHs) based on diet, indoor and outdoor activities; MN = micronuclei; PAHs = Polycyclic aromatic hydrocarbons; Occup_PAHs = (years of work in the cokery) × (1-pyrenol in µmoles/mol creatinine)

**Fig. 2:**
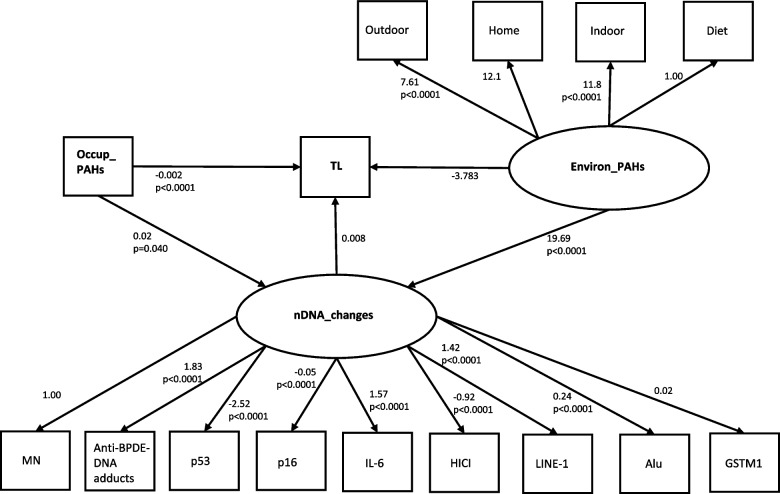
Path diagram of mediation model for TL. Path diagram of a mediation model showing the dependent variable «TL», the mediator variable «nDNA_changes» and two independent variables «Occup_PAHs» and «Environ_PAHs». An oval indicates the latent variable, square boxes indicate the observed variables, arrows specify the direction of causal flow, an arrowed route is a path, and the estimated beta coefficients with *p*-values appeared along the paths. The effect of one variable on another is called direct. Paths from each latent variable (nDNA_changes, Environ_PAHs) to each endogenous variable are also drawn. Abbreviations: TL = telomere length; nDNA_changes = Latent variable estimated by SEM (overall change of DNA) based on MN, Anti-BPDE-DNA adducts, p53, p16, IL-6, HICI, LINE-1, Alu, GSTM1; Environ_PAHs = Latent variable estimated by SEM (environmental exposure to PAHs) based on diet, home, indoor and outdoor activities; MN = micronuclei; PAHs = Polycyclic aromatic hydrocarbons; Occup_PAHs = (years of work in the cokery) × (1-pyrenol in µmoles/mol creatinine)

**Fig. 3:**
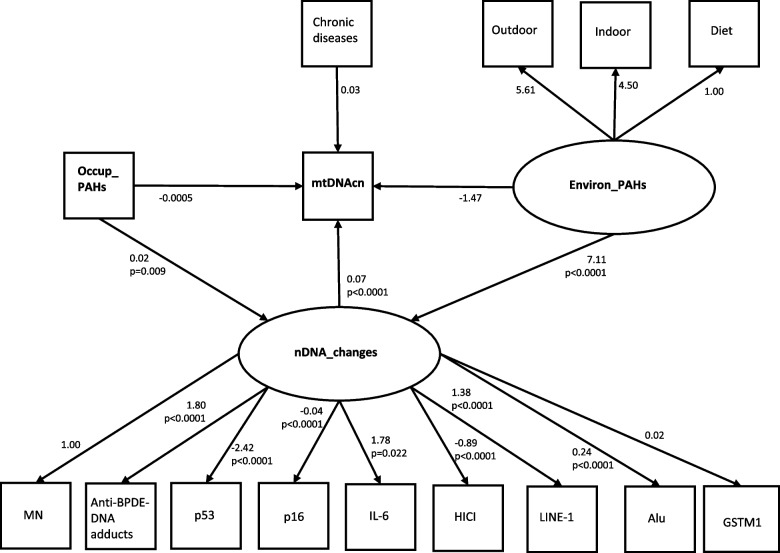
Path diagram of mediation model for mtDNAcn. Path diagram of a mediation model showing the dependent variable «mtDNAcn», the mediator variable «nDNA_changes» and two independent variables «Occup_PAHs» and «Environ_PAHs». An oval indicates the latent variable, square boxes indicate the observed variables, arrows specify the direction of causal flow, an arrowed route is a path, and the estimated beta coefficients with p-values appeared along the paths. The effect of one variable on another is called direct. Paths from each latent variable (nDNA_changes, Environ_PAHs) to each endogenous variable are also drawn. Abbreviations: mtDNAcn = mitochondrial DNA copy number; nDNA_changes = Latent variable estimated by SEM (overall change of DNA) based on MN, Anti-BPDE-DNA adducts, p53, p16, IL-6, HICI, LINE-1, Alu, GSTM1; Environ_PAHs = Latent variable estimated by SEM (environmental exposure to PAHs) based on diet, indoor and outdoor activities; MN = micronuclei; PAHs = Polycyclic aromatic hydrocarbons; Occup_PAHs = (years of work in the cokery) × (1-pyrenol in µmoles/mol creatinine)

Independent, dependent and mediator variables and their relationships can be represented as a graph formed by vertices and edges, where vertices display variables and the relationships between them take the form of lines (or edges) going from one vertex to another. These edges are directed, which means that they have a single arrowhead indicating their effect. A DAG is also acyclic, which means that there are no feedback loops such that following those directions will never form a closed loop. DAG is more specifically concerned with structural causal models displaying causal assumptions about a set of variables. Figures 1, 2 and 3 display three DAGs with a similar polygon: two triangles sharing one edge. The vertices are Occup_PAHs, DNAmAge (or TL or mtDNAcn) and nDNA_changes on the left triangle, Environ_PAHs, DNAmAge (or TL or mtDNAcn) and nDNA_changes on the right triangle. The arrows specify direct effects and indirect path. The latter is an effect of PAHs mediated through nDNA_changes.

iii) Structural equation models (SEM)

Another way to think about DAGs is as non-parametric structural equation models. Therefore, all the above assumptions were converted into three models of structural equation modeling (SEM), one for each final outcome (DNAmAge, TL or mtDNAcn). In the first and second set of parentheses of STATA commands we specified the estimations of the two latent variables: Environ_PAHs (an independent variable); and nDNA_changes (the mediator variable). In the third and fourth sets of parentheses consist of fitting two regression models: the first regression is of nDNA_changes on the two PAHs exposures generated by occupational or environmental sources; the second regression is the model for the final outcome (either DNAmAge, TL, mtDNAcn). Notice that “nDNA_changes” was a dependent variable in the first regression (third set) and an explanatory variable in the second regression (fourth set of parentheses). The exposure coefficient in the second model of regression that includes the mediator is then generally taken as a measure of the direct effect because the effect on the outcome appears to remain even when control has been made for the mediator. The ending command vce(cluster occup) specifies how the VCE (variance–covariance matrix of the estimators) and the standard errors reported by SEM were calculated. As already reported, the study population consisted of 49 blue-collar workers (employed in 3 Polish coking plants) and 45 white-collar workers (office workers of the Institute of Occupational Medicine and Environmental Health in Sosnowiec). Therefore, it would not be unreasonable to assume that the error of one person is correlated with those of others belonging to the same group because each social class tends to be homogeneous. In such cases, where the observations are correlated and the cluster is known, we typed: vce(cluster occup).

According to the above description, the STATA command syntax was the following.

1. sem (Environ_PAHs -> diet indoor outdoor) (nDNA_changes -> mn adducts p53 p16 il6 hici line alu gstm) (nDna_changes <- Environ_PAHs Occup_PAHs) (DNAmAge <- chron_d nDNA_changes Environ_PAHs Occup_PAHs), vce(cluster occup).
2. sem (Environ_PAHs -> diet indoor outdoor home) (nDNA_changes -> mn adduct p53 p16 il6 hici line alu gstm) (nDNA_changes <- Environ_PAHs Occup_PAHs) (TL <- nDNA_changes Environ_PAHs Occup_PAHs), vce(cluster occup).
3. sem (Environ_PAHs -> diet indoor outdoor) (nDNA_changes -> mn adduct p53 p16 il6 hici line alu gstm) (nDNA_changes <- Environ_PAHs Occup_PAHs) (mtDNAcn <- chron_d nDNA_changes Environ_PAHs Occup_PAHs), vce(cluster occup).

Some variables, particularly those with dichotomous values, were removed because they prevented SEM algorithm to converge.

We used three SEM goodness-of-fit statistics: (1) Standardized root mean squared residual (SRMR); (2) the coefficient of determination (CD); and (3) Stability index. Tables 2, 3 and 4 show the three groups of SEM results (Structural Equations, Measurement, Covariates). Structural equations include the beta coefficients (with a “minus” sign indicating an inverse relationship), 95% confidence intervals (95%CI) and *p*-values for each of two structural equation models. Measurement including the beta coefficients for this measurement model can be interpreted as correlation coefficients describing the direction (positive or negative) and degree (strength) of the relationship between each indicator and the latent variables Environ_PAHs and nDNA_changes. Covariances representing set of concurrent regression equations to yield coefficient estimators and the covariance is a measure of cross-equation correlation. All coefficients specifying the effects are expressed in the own variable’s scale of units.

SEM results were also presented graphically (Figs. 1, 2 and 3) using the graphical interface of SEM. In Figs. 1, 2 and 3, square boxes stand for variables, arrows specify the direction of causal flow, an arrowed route is a path. The estimated beta coefficients with corresponding p-values appeared along the paths. The figures are a useful synthesis of the findings.

iv) Study size

The sample size required for SEM is dependent on model complexity, the estimation method used, and the distributional characteristics of observed variables. The best option is to consider the model complexity (i.e., the number of exogenous variables) and the following rules of thumb: minimum ratio 5:1, with a recommended ratio of 10:1, or a recommended ratio of 15:1 for data with no normal distribution [36, 37]. With three exogenous variables (occupational exposure to PAHs, environmental exposure to PAHs and chronic diseases) used in the SEM model, we should have a minimum of 45 (= 15 × 3) subjects; in total we reached 87 with complete data, thus fulfilling these requirements.

e) Latent variables

A numerical value for each latent variable (nDNA_changes and Environ_PAHs) was estimated by SEM program for each subject. Multiple summary statistics were calculated conditioned on a categorical variable that identified the two groups: coke-oven workers and controls. The numerical statistics (median, min, max) were reported in Table 1, together with the p-value of Wilcoxon rank sum test for the equality of the median distribution across the two groups.

**Table 1:** Main characteristics of polish male coke-oven workers and non-exposed controls

Interval variables *Frequency variables*	Median(min; max) *Number(%)*		Statistical tests and p-values^@^
	Coke-oven workers n=49	Controls n=45	
**General characteristics and PAHs**^**§**^**exposure**			
Age (Years)	36(20−59)	37(21−58)	0.911
Diet^a^	*8(16%)*	*8(18%)*	*0.852*
Fruit and vegetables^b^	*27(55%)*	*22(49%)*	*0.549*
Indoor PAH exposure^c^	*26(53%)*	*18(40%)*	*0.205*
Outdoor PAH exposure^d^	*17(34%)*	*16(36%)*	*0.930*
Nicotine & metabolites (mg/mmol creatinine)^e^	*0(0%)*	*0(0%)*	
Former smokers	*20(41%)*	*16(36%)*	*0.600*
Years since cessation	*6(1-26)*	*7(1-20)*	*0.640*
Length of work in the cokery (years)	10.5 (1; 40.0)	0 (0; 0)	
1-pyrenol (μmoles/mol creatinine) ^f^	3.1(0.4; 7.5)	0.1(0.0; 0.4)	**0.00001**
Occup_PAHs^g^	36.2(3.6; 253.0)	0(0; 0)	**0.00001**
Environ_PAHs^h^	0.01313(-0.0471; 0.0607)	-0.0131(-0.0970; 0.0378)	**0.0001**
nDNA_changes^i^	1.7377(-0.0006; 4.0372)	-0.5360 (-1.0397; 0.9357)	**0.00001**
**Genotoxic alterations**			
Anti-BPDE-DNA adducts (adducts/10^8^ nucleotides) ^k^	5.1 (0.9; 12.2)	0.1 (0.1; 5.6)	**0.00001**
MN (MN/1000BN cells)	4 (1.0; 11.0)	0.01 (0; 4.0)	**0.00001**
**Epigenetic alterations**			
LINE-1 (%mC)	80.3(56.91; 83.7)	75.7(55.7; 85.7)	**0.00001**
p53 (%mC)	11.8(5.6; 25.1)	18.6(6.9; 46.2)	**0.0003**
Alu (%mC)	23.6(21.8; 24.5)	22.9(20.6; 24.4)	**0.0007**
HICI (%mC)	17.2(10.4; 33.0)	20.4(7.4; 34.4)	**0.048**
IL-6 (%mC)	48.4(34.5; 69.9)	44.7(28.7; 61.8)	0.095
p16 (%mC)	1.9(1.3; 3.6)	2.1(0.8; 3.9)	0.205
**GSTM1 genotyping**			
*0/*0	*18(41%)*	*25(54%)*	0.204
*1/*1 and *0/*1	*26(59%)*	*22(46%)*	
**Outcomes**			
DNAmAge ^l^	37(17.0; 54.0)	36(19.0; 52.0)	0.567
AgeAcc ^m^	-1(-22.0; 15.0)	-3(-13.0; 4.0)	0.096
Subjects with AgeAcc ≥ 0 N (%)	*20(40.8%)*	*7(15.6%)*	**0.007**
TL ^n^	1.0(0.3; 3.0)	1.2(0.4; 2.1)	**0.038**
mtDNAcn^o^	1.0(0.4; 2.5)	0.9(0.3; 1.7)	0.128

^@^ Statistical tests: Wilcoxon rank-sum test for interval variables and Chi-square test for frequency variables

^§^ PAHs = Polycyclic Aromatic Hydrocarbons

^a^ Charcoaled meat consumption ≥ once a week

^b^ Daily consumption of fruit or vegetables

^c^ Wood or coal-based heating at home

^d^ High environmental exposure from residence in town, intense traffic and presence of industries near home

^e^ Positive subjects with values higher than threshold limit of assay (0.01 mg/mmoles creatinine)

^f^ PAHs exposure evaluated by urinary excretion of 1-pyrenol

^g^ Occup_PAHs = (years of work in the cokery) × (1-pyrenol in µmoles/mol creatinine)

^h^ Environ_PAHs = Latent variable estimated by SEM (environmental exposure to PAHs) based on diet, indoor and outdoor activities

^i^ nDNA_changes = Latent variable estimated by SEM (overall change of DNA) based on MN, Anti-BPDE-DNA adducts, LINE-1, p53, Alu, HICI, IL-6, p16, GSTM1

^k^ A value of 0.125 adducts/10^8^ nucleotides was assigned to subjects with non-detectable adducts

^l^ DNAmAge = DNA methylation age

^m^ AgeAcc = difference between the DNAmAge and the chronological age

^n^ TL = Telomere length in lymphocytes

^o^ mtDNAcn = mitochondrial DNA copy number in lymphocytes

The analysis was conducted with the statistical package STATA 14.

## Results

### Descriptive results

Table 1 shows the main characteristics of Polish coke-oven workers and non-exposed controls. We used the Wilcoxon rank-sum test to compare interval variables and Chi-square test for frequency variables; the corresponding p-values are reported in the last column of Table 1. To reduce confounding factors, all participants were males and noncurrent smokers (either never-smokers or former smokers who had quit smoking at least 1 year before sample collection as confirmed by analysis of nicotine and its metabolites) and matched for age (ages of two groups were almost overlapping and the difference was not significant) and ethnicity. Furthermore number of former smokers and years since cessation were equally distributed between workers and controls. The prevalence of indoor PAH exposure was higher, but non-significant, among coke-oven workers while that of diet and outdoor PAH exposure was similar in both groups. The cumulative occupational exposure Occup_PAHs was highly significant (*p* = 0.00001) in coke-oven workers compared to control subjects, for whom it was always zero, as well as the urinary excretion of 1-pyrenol (*p* = 0.00001). The latent variable Environ_PAHs, based on arbitrary units, had a negative median indicating lower PAHs exposure among controls and a positive median value suggesting higher exposure to environmental PAHs among coke-oven workers (*p* = 0.0001). Highly significant differences among groups were observed for anti-BPDE–DNA adducts (*p* = 0.00001), micronuclei (*p* = 0.00001), LINE-1 (*p* = 0.00001), p53 (*p* = 0.0003) and Alu (*p* = 0.0007), but not for other variables used as indicators to infer nDNA_changes. The median of this score, based on arbitrary units, was negative among controls and positive among coke-oven workers, suggesting that the nuclear DNA was more “damaged” among the latter than the formers; the difference was highly significant (*p* = 0.00001). No difference in the distribution of GSTM1 genotyping was found. In univariate analysis biological aging determined by DNAmAge, TL and mtDNAcn showed that the percentage of subjects with AgeAcc ≥ 0 (biological age older than chronological age) was higher in coke-oven workers than controls (*p* = 0.007), and TL is significantly shorter in coke-oven workers than in controls (*p* = 0.038), while mtDNAcn was similar in the two groups. In total 6 out of 49 (10%) coke-oven workers and 10 out of 45 (18%) controls declared diabetes or hypertension, no participant had cancer disease. Furthermore, no correlation between years since cessation, in former smokers in both coke-oven workers and controls, and biological aging detected by AgeAcc (*r* = -0.134, *p* = 0.407 and *r* = -0.062, *p* = 0.666), TL (*r* = -0.006, *p* = 0.654 and *r* = -0.017, *p* = 0.111) and mtDNAcn (*r* = 0.003, *p* = 0.691 and *r* = -0.006, *p* = 0.726), was found.

### Outcome results

#### DNAmAge

Table 2 shows three groups of SEM results (Structural Equations, Measurement, Covariates) for the mediation analysis for DNAmAge. In “Structural Equations”, the first model reveals that DNAmAge significantly associated with Chronic diseases (*p* < 0.0001) and Occup_PAHs (*p* < 0.0001), but not with Environ_PAHs (*p* = 0.267) or nDNA_changes (*p* = 0.079). The second model reveals that nDNA_changes significantly increased with Environ_PAHs (*p* = 0.008), while Occup_PAHs was not significant (*p* = 0.094). In the section “Measurement” the latent variable nDNA_changes was positively correlated with Anti-BPDE-DNA adducts (*p* < 0.0001), LINE-1 (*p* < 0.0001), Alu (*p* < 0.0001) and IL-6 (*p* = 0.005); this correlation was negative with p53 (*p* < 0.0001), HICI (*p* < 0.0001) and p16 (p<0.0001). In the section “Covariances” the findings demonstrated that chronic disease was individually correlated with Environ_PAHs (*p* = 0.033). Using the graphical interface of SEM, the results shown in Table 2 were displayed as a path diagram in Fig. 1.

**Table 2:** SEM results of mediation analysis for DNAmAge

	Endogenous variables	Exogenous variables	Beta Coef.	95%CI		*p*-value
				Lower	Upper	
Structural Equations	DNAmAge	Occup_PAHs	0.07	0.06	0.08	**0.000**
		Environ_PAHs	38.6	3.40	73.9	0.267
		nDNA_changes	−0.98	−2.08	0.11	0.079
		Chronic diseases	4.44	2.83	6.06	**0.000**
	nDNA_changes	Occup_PAHs	0.02	−0.003	0.05	0.094
		Environ_PAHs	4.28	1.14	7.42	**0.008**
Measurement	Diet ← Environ_PAHs		1	(constrained)		
	Indoor ← Environ_PAHs		3.43	−7.11	14.0	0.523
	Outdoor ← Environ_PAHs		7.86	−5.32	21.0	0.242
	MN ← nDNA_changes		1	(constrained)		
	Anti-BPDE-DNA adducts ← nDNA_changes		1.81	1.63	1.99	**0.000**
	LINE-1 (%mC) ← nDNA_changes		1.42	1.24	1.61	**0.000**
	p53 (%mC) ← nDNA_changes		−2.53	−2.97	−2.10	**0.000**
	Alu (%mC) ← nDNa_changes		0.25	0.22	0.27	**0.000**
	HICI (%mC) ← nDNA_changes		−0.96	−1.28	−0.63	**0.000**
	IL-6 (%mC) ← nDNA_changes		1.66	0.49	2.83	**0.005**
	p16 (%mC) ← nDNA_changes		−0.05	−0.06	−0.04	**0.000**
	GSTM1 ← nDNA_changes		0.02	-0.12	0.21	**0.578**
Covariances	cov(Occup_PAHs,Environ_PAHs)		0.54	-1.01	2.14	0.483
	cov(Chron_d,Environ_PAHs)		−0.01	−0.02	−0.001	**0.033**

Three groups of SEM results (structural equations; measurement; covariances) for the mediation analysis of biological age. Beta coefficients with “minus” sign indicating inverse relationship. Lower and upper limit of 95% confidence intervals (95%CI) and p-values estimated with standard error adjusted for 2 clusters (coke-oven workers and controls). Number of observations = 87

*Abbreviations*: *DNAmAge* DNA methylation age, *nDNA_changes* Latent variable estimated by SEM (overall change of DNA) based on MN, Anti-BPDE-DNA adducts, LINE-1, p53, Alu, HICI, IL-6, p16, GSTM1, *Environ_PAHs* Latent variable estimated by SEM (environmental exposure to PAHs) based on diet, indoor and outdoor activities, *MN* Micronuclei, *PAHs* Exposure to Polycyclic aromatic hydrocarbons, *Occup_PAHs* (years of work in the cokery) × (1-pyrenol in μmoles/mol creatinine)

Goodness of fit statistics: Standardized root mean squared residual (SRMR) = 0.09; Coefficient of determination (CD) = 0.703; Stability index = 0 (SEM satisfies stability condition)

#### TL

Table 3 reports three groups of SEM results (Structural Equations, Measurement, Covariates) for the mediation analysis for TL. In “Structural Equations”, the first model shows that TL significantly decreased with Occup_PAHs (*p* < 0.0001), but not with Environ_PAHs (*p* = 0.074) or nDNA_changes (*p* = 0.966). The second model reveals that nDNA_changes significantly increased with Environ_PAHs (*p* < 0.0001) and Occup_PAHs (*p* = 0.040). In “Measurement”, the first model indicated that the most significant determinants of environmental PAH exposure were “indoor” (*p* < 0.0001) and “outdoor” (*p* < 0.0001). Their positive coefficients indicate that the latent variable Environ_PAHs tends to increase with increasing values of these variables. In the second measurement model, the results of Table 3 were equal to those shown in Table 2, that the latent variable nDNA_changes tends to increase with rising values of the methylation states (%) of LINE-1, Alu and IL-6 (*p* < 0.0001), and of anti-BPDE–DNA adducts (*p* < 0.0001) that are the positive coefficients. While, the negative coefficient of the methylation state (%) of p53, HICI, and p16 (*p* < 0.0001) means that these factors tend to go in the opposite direction and nDNA_changes (latent variable) increases with their decreasing values. No significant result was obtained for the relationship between Occup_PAHs and Environ_PAHs (*p* = 0.683). Using the graphical interface of SEM, the results shown in Table 3 were displayed as a path diagram in Fig. 2.

**Table 3:** SEM results of mediation analysis for TL

	Endogenous variables	Exogenous variables	Beta Coef.	95%CI		*p*-value
				Lower	Upper	
Structural Equations	TL	Occup_PAHs	−0.002	−0.003	−0.001	**0.000**
		Environ_PAHs	−3.783	−7.928	0.363	0.074
		nDNA_changes	0.008	−0.04	0.04	0.966
	nDNA_changes	Occup_PAHs	0.021	0.002	0.040	**0.040**
		Environ_PAHs	19.69	12.16	27.23	**0.000**
Measurement	Diet ← Environ_PAHs		1	(constrained)		
	Indoor ← Environ_PAHs		7.608	5.466	9.750	**0.000**
	Outdoor ← Environ_PAHs		11.78	7.098	16.47	**0.000**
	Home ← Environ_PAHs		12.09	−1.024	25.20	0.071
	MN← nDNA_changes		1	(constrained)		
	Anti-BPDE-DNA adducts ← nDNA_changes		1.829	1.429	2.229	**0.000**
	LINE-1 (%mC) ←nDNA_changes		1.417	1.405	1.429	**0.000**
	p53 (%mC) ← nDNA_changes		−2.519	−2.624	−2.414	**0.000**
	Alu (%mC) ← nDNA_changes		0.235	0.208	0.261	**0.000**
	HICI (%mC) ← nDNA_changes		−0.915	−1.133	−0.697	**0.000**
	IL-6 (%mC) ← nDNA_changes		1.572	0.824	2.320	**0.000**
	p16 (%mC) ← nDNA_changes		-0.052	-0.057	-0.048	**0.000**
	GSTM1 ← nDNA_changes		0.016	-0.031	0.063	0.504
Covariances	cov(Occup_PAHs,Environ_PAHs)		0.122	-0.463	0.706	0.683

Three groups of (structural equations, measurement and covariances) for the mediation analysis; standardized beta coefficients (with “minus” sign indicating inverse relationship) with lower and upper limit of 95% confidence intervals (95%CI) and p-values. Standard Error adjusted for 2 clusters (coke-oven workers and controls). Number of observations = 87

*Abbreviations*: *TL* Telomere length, *Chronic_d* Chronic diseases, *nDNA_changes* Latent variable estimated by SEM (overall change of DNA) based on MN, Anti-BPDE-DNA adducts, LINE-1, p53, Alu, HICI, IL-6, p16, GSTM1, *Environ_PAHs* Latent variable estimated by SEM (environmental exposure to PAHs) based on diet, features ofhome, indoor and outdoor activities, *MN* Micronuclei, *PAHs* Exposure to Polycyclic aromatic hydrocarbons, *Occup_PAHs* (years of work in the cokery) × (1-pyrenol in μmoles/mol creatinine)

Goodness of fit statistics: Standardized root mean squared residual (SRMR) = 0.092; Coefficient of determination (CD) = 0.914; Stability index = 0 (SEM satisfies stability condition)

#### mtDNAcn

Table 4 shows three groups of SEM results (Structural Equations, Measurement, Covariates) for the mediation analysis for mtDNAcn. In “Structural Equations”, the first model shows that mtDNAcn significantly increased with nDNA_changes (*p* < 0.0001), but not with Occup_PAHs (*p* = 0.598) or Environ_PAHs (*p* = 0.719) or chronic diseases (*p* = 0.294). The second model reveals that nDNA_changes significantly increased with Environ_PAHs (*p* < 0.0001) and Occup_PAHs (*p* = 0.009). In “Measurement” section, the most significant indicators for the latent variable nDNA_changes are the methylation states (%) of *LINE*-1 and Alu (*p* < 0.0001) and IL-6 (*p* = 0.022), the presence of anti-BPDE–DNA adducts (*p* < 0.0001), that are positively correlated, and the methylation state (%) of *p53,* HICI and p16 (*p* < 0.0001), which are negatively correlated. In “Covariances” section, no significant result was obtained for the relationship between Occup_PAHs and Environ_PAHs (p = 0.688) as well as between Chronic diseases and Occup_PAHs (*p* = 0.420). Using the graphical interface of SEM, the results shown in Table 4 were displayed as a path diagram in Fig. 3.

**Table 4:** SEM results of mediation analysis for mtDNAcn

	Endogenous variables	Exogenous variables	Beta Coef.	95%CI		*p*-value
				Lower	Upper	
Structural Equations	mtDNAcn	Occup_PAHs	-0.0005	-0.0022	0.0013	0.598
		Environ_PAHs	-1.4688	-9.4714	6.5340	0.719
		nDNA_changes	0.0690	0.0483	0.0896	**0.000**
		Chronic diseases	0.0338	-0.0293	0.0969	0.294
	nDNA_changes	Occup_PAHs	0.0214	0.0054	0.0373	**0.009**
		Environ_ PAHs	7.1108	6.0885	8.1331	**0.000**
Measurement	Diet ← Environ_ PAHs		1	(constrained)		
	Indoor ← Environ_ PAHs		4.4960	-2.2258	1.1217	0.190
	Outdoor ← Environ_ PAHs		5.6125	-29.545	40.771	0.754
	MN← nDNA_changes		1	(constrained)		
	Anti-BPDE-DNA adducts ← nDNA_changes		1.8001	1.6520	1.9483	**0.000**
	LINE-1 (%mC) ← nDNA_changes		1.3820	1.1354	1.6285	**0.000**
	p53 (%mC) ← nDNA_changes		-2.4248	-3.0079	-1.8417	**0.000**
	Alu (%mC) ← nDNA_changes		0.2372	0.2365	0.2379	**0.000**
	HICI (%mC) ← nDNA_changes		-0.8902	-1.1999	-0.5805	**0.000**
	IL-6 (%mC) ← nDNA_changes		1.7827	0.2553	3.3102	**0.022**
	p16 (%mC) ← nDNA_changes		-0.0409	-0.0557	-0.0261	**0.000**
	GSTM1 ← nDNA_changes		0.0241	-0.0122	0.0606	0.193
Covariances	cov(Occup_PAHs, Environ_PAHs)		0.2981	-115.55	1.7517	0.688
	cov(Chron_d,Environ_ PAHs)		-0.0096	-0.0329	0.0137	0.420

Three groups of SEM results (structural equations, measurement and covariances) for the mediation analysis; standardized beta coefficients (with “minus” sign indicating inverse relationship) with lower and upper limit of 95% confidence intervals (95%CI) and p-values. Standard Error adjusted for 2 clusters (coke-oven workers and controls). SEM’s goodness-of-fit statistics at bottom of table. Number of observations = 87

*Abbreviations*: *mtDNAcn* Mitochondrial DNA copy number, *nDNA_changes* Latent variable estimated by SEM (overall change of DNA) based on MN, Anti-BPDE-DNA adducts, LINE-1, p53, Alu, HICI, IL-6, p16, GSTM1, *MN* Micronuclei, *PAHs* Exposure to Polycyclic aromatic hydrocarbons, *Environ_PAHs* Latent variable estimated by SEM (environmental exposure to PAHs), *Occup_PAHs* (years of work in the cokery) × (1-pyrenol in μmoles/mol creatinine)

Goodness of fit statistics: Standardized root mean squared residual (SRMR) = 0.092; Coefficient of determination (CD) = 0.914; Stability index = 0 (SEM satisfies stability condition)

## Discussion

The present study evaluated by SEM analysis the relative magnitude of different pathways by which PAHs exposure may affect the main hallmarks of biological aging – DNAmAge, TL, and mtDNAcn– supposing that some of the effects of environmental and occupational exposure to PAHs also act indirectly by triggering nDNA alterations. Our study reveals that DNAmAge increased with occupational PAH exposure and the presence of chronic diseases; but not with environmental PAH exposure and nDNA alterations. On the same line, TL is confirmed to be directly decreased with occupational PAH exposure; but not with environmental PAH exposure and nDNA alterations. Conversely, mtDNAcn indirectly increased with environmental and occupational PAH exposure acting through nDNA alterations.

The direct positive relationship between DNAmAge and occupational PAH exposure is in line with a previous study by Li et al. [38], underscoring the negative impact of high PAH exposure on aging. The work by Li et al. [38] was however performed with a different methylation age predictor, specifically built for Chinese populations, and reported that a 1-unit increase in 1-hydroxypyrene, even deriving from smoking behavior, was associated with a 0.53-y increase in AgeAcc. In our work, all study subjects, all non-current smokers and exposed individuals were similar to controls for age, gender and ethnicity minimizing the possibility that the higher biological aging could depend on factors other than PAH exposure. In addition, we evaluated several other potential confounding factors, including dietary PAHs, indoor and outdoor PAH exposures that showed no differences between the two groups. DNA methylation is currently the most promising molecular marker for monitoring biological aging and predicting life expectancy [39]. In humans, DNA methylation changes start early in life, as demonstrated by longitudinal studies of infants' blood [40, 41]. Notably, these early epigenetic profiles continue to accumulate changes with the advancement of age as shown in twins that do not share the same habits and/or environments [42, 43], indicating aging-associated DNA methylation changes depending on environmental factors. In our previous study [23], DNAmAge and AgeAcc blood lymphocytes correlated with those of pulmonary cells, advising that blood lymphocytes could be a validate surrogate tissue for lung aging studies. This suggests that the PAH-related acceleration aging of blood lymphocytes observed in coke-oven worker mirrors what happens in the respiratory tract.

Also we found an increase in DNAmAge related to chronic diseases. This finding is consistent with previous studies that reported a substantial increase in DNAmAge associated with age-related chronic diseases including frailty [44], cancer [45], diabetes [46], cardiovascular diseases (CVD) [47], dementia [48], as well as with chronic obstructive pulmonary disease (COPD) [23]. The latter, from our previous work [23], shows that blood leukocytes DNAmAge and AgeAcc significantly increase (become older) in COPD patients, and with a reduction in lung function (FEV1%). DNAmAge, therefore, seems an accountable signature of the epigenetic aging chronic disease-related.

The direct negative relationship, detected by SEM analysis, between TL and PAH occupational exposure, not influenced by chronic diseases, confirmed the results of our previous study in coke-oven workers showing that telomeres significantly reduced with years of work [12]. The fact that the environmental exposure to PAHs in which the most significant determinants were “indoor” and “outdoor” are not directly correlated depends on the fact that the occupational exposure of coke-oven workers is very high. In our study, the cumulative occupational exposure to PAHs was in fact very much higher in workers compared to controls, given that the majority of the PAH-exposed workers exceeded the Biological Exposure Index proposed by Jongeneelen [49] for urinary 1-pyrenol. The direct negative relationship between TL and PAHs was also observed in everyday-life exposure to PAHs in the general population [27].

Telomeres, repetitive functional complexes of DNA/protein at the ends of chromosomes, preserve DNA integrity that in their absence would be gradually lost with each cell division [50]. Their length measured in blood lymphocytes is considered an indicator of biological aging [50]. Loss of telomere sequence in lymphocytes has been also related to adverse age-related outcomes, in particular CVD [51, 52] and respiratory diseases [53]. Exposure to PAHs may pose a risk not only for lung cancer, but also for CVD, including atherosclerosis, hypertension, thrombosis and myocardial infarction [54]. Since PAH exposure is pervasive and modifiable, it is an appropriate target for age-related disorders, especially CVD, prevention research studies.

The chances that the association with shorter TL could depend on factors other than PAH exposure were minimized because all study subjects were non-current smokers and exposed individuals were similar to controls for age, gender and ethnicity. Furthermore, TL was not affected by chronic diseases.

MtDNAcn indirectly increased with environmental and occupational PAH exposure, acting through nDNA_changes. nDNA_changes is the latent variable estimated by SEM (overall change of DNA) in which adducts, LINE-1, p53, Alu, HICI, IL6, p16, are major determinants. This would suggest a relationship between the number of changes in nDNA and mtDNA. This result confirmed our previous study detecting a significantly higher mtDNAcn in coke-oven workers using another statistical analysis [13]. This observation is in line with previous findings by Sahin and colleagues [55] that showed a potential unifying mechanism connecting the nucleus and mitochondria in cellular aging. In that work, progressive nuclear changing, mediated by the activation of a p53-dependent pathway, was found to determine a reduction of mitochondrial function and mtDNAcn [55].

The present study has several strengths. The enrollment of the study participants was carefully designed to minimize potential confounding and increase the capability to reveal PAH effects by selecting non–current smoking males, all coke-oven workers and controls living in the same residential area, reducing the probability that the observed associations were dependent on factors other than occupational PAH exposure. We also evaluated several other potential sources of PAH exposure, including dietary PAHs, indoor and outdoor PAH exposures, which showed no differences between coke-oven workers and controls. Our study had reliable measurements of PAH(B[a]P) internal and target doses. Also, we measured in the study participants, biomarkers of genetic instability and methylation that allowed for characterizing the inter-correlation between nDNA and mtDNAcn alterations. Furthermore, we applied the method proposed by Zbieć-Piekarska et al. [19] to assess DNAmAge, on data from five CpG sites using the locus-specific technology pyrosequencing with some modifications [23, 32]. This model, based on an algorithm developed in a larger sample (*n* = 420) and then validated in a smaller one (*n* = 300) covering the entire adult life span, shows that DNAmAge highly correlates (*r* = 0.94) to chronological age with a mean deviation (4.5 years) similar to those of Horvath [17] and Hannum et al. [18] (*r* = 0.96 and *r* = 0.91) with 3.6 and 4.9 years mean, which are considered the reference methods. We recently automated this method to improve efficiency and speed while maintaining high prediction accuracy [23, 32]. By using this method, we can perform the analyses in a standardized manner reducing errors, and this is another strong point of our study. Moreover, it is interesting to note that pyrosequencing has the potential for multiplexing, which can simplify the protocol and reduce the cost of technical analysis. Finally, the results of this study appear to be biologically plausible and the direction of the effects is consistent with the available literature data on aging mechanisms.

We also recognize limitations to our study. This is a small-sized study and its results need to be confirmed in a larger independent investigation. Its cross-sectional design does not allow for investigating the temporal relationship of PAH exposure with biomarkers of damage, genetic instability, and indicators of biological aging. The absence of air monitoring, as well as repeated biological sampling, are also limitations of the study exposure assessment strategy. However, PAH exposure was assessed using biomarkers of internal dose (urinary 1-pyrenol) and target dose (anti-BPDE–DNA adduct), which may more appropriately represent the effective exposure dose. We also recognize that socio-economic factors, in particular related to education and income, were not part of this study. We cannot therefore exclude that different socio-economic status in particular related to education and income, might have contributed, along with PAH exposure in the increased of biological aging observed in lymphocytes of coke-oven workers. Socio-economic factors are however non-specific factors indirectly linked to lifestyle habits such as cigarette smoking or diet, environmental exposures, housing, which we have considered in our study. The enrollment of the study participants was in fact carefully designed to minimize potential confounding factors. We matched coke-oven workers and controls for their individual characteristics, including age, gender, and ethnicity. Furthermore, the lack of statistical significant differences in outdoor and indoor exposures as well as the fact that all participants live in the same residential area, lead us to assume that they could be exposed to similar levels of environmental factors different from PAHs. In addition, we adjusted the analysis contrasting high-exposed workers, as well as those based on continuous exposure or biomarker variables, for age.

The attractiveness of SEM analysis stems mainly from the fact that researchers have recognized the necessity of grasping the complex interrelations between multiple variables under study. Traditional statistical approaches apply solely to a limited number of variables and thus fail to deal with emerging sophisticated theories. SEM analysis is a statistical technique that links observed data with qualitative causative assumptions and tests whether variables are interdependent, and if so, the details of their interactions. This is achieved through an estimation procedure [56], which uses a set of concurrent regression equations to yield coefficient estimators more efficiently than single-equation estimators. This methodology is appropriate for the investigation of complex interrelationships, as it tests causative relationships instead of mere correlations [24].

## Conclusion

We showed that DNAmAge was positively and significantly correlated with occupational PAH exposure. Our findings indicate that occupational PAH exposure makes coke-oven workers biologically older. We also confirmed that TL was negatively correlated with occupational PAH exposure, suggesting that both mechanisms of biological aging (DNAmAge and TL) are PAH targets. Also, differences in mtDNAcn, which is typically altered through nDNA alterations triggered by environmental and occupational PAH exposure, suggested a nuclear-mitochondrial core-axis of aging. Lowering PAH exposure may prevent biological aging and age-related diseases.

## Data Availability

Data will be available if required.

## Abbreviations

AgeAcc: Age acceleration
Anti-BPDE–DNA adducts: Anti-benzo[a]pyrene diolepoxide adducts
CVD: Cardiovascular diseases
DAG: Directed acyclic graph
DNAmAge: Dna methylation age
mtDNAcn: Mitochiondrial DNA copy number
PAHs: Polycyclic aromatic hydrocarbons
qPCR: Quantitative Polymerase chain reaction
SEM: Structural equation models
TL: Telomere length
